# In-Game Need Satisfaction, Frustration, and Gaming Addiction Patterns Across Subgroups of Adolescents Through Structural Equation Modeling: Cross-Sectional and Instrument Validation Study of the Youth Gaming Experience Scales

**DOI:** 10.2196/63612

**Published:** 2025-11-12

**Authors:** Amparo Luján-Barrera, Lydia Cervera-Ortiz, Mariano Chóliz

**Affiliations:** 1 Department of Basic Psychology Faculty of Psychology University of Valencia (UV) Valencia Spain

**Keywords:** self-determination theory, adolescence, in-game need satisfaction, scale validation, basic psychological needs, structural equation modeling, game addiction, psychological needs, Spain

## Abstract

**Background:**

Gaming is a prevalent activity during adolescence, a developmental stage characterized by vulnerability to gaming disorder (GD). According to the self-determination theory, gaming environments can satisfy and frustrate basic psychological needs—autonomy, competence, and relatedness—processes linked to GD. However, existing research has primarily focused on adult populations, and validated instruments assessing both in-game need satisfaction (NS) and in-game need frustration (NF) in adolescents are lacking.

**Objective:**

This study aimed to address this gap by validating the Spanish version of the Basic Psychological Need Satisfaction and Frustration Scale for Gaming (BPNSFS-G) in adolescents. We examined its psychometric properties, its relationship with GD and gaming behavior, and differences in NS and NF across subgroups defined by risk factors for GD (sex, the developmental stage, and the gaming modality).

**Methods:**

A total of 1174 adolescents (mean 12.07, SD 1.23; middle adolescents: 815/1174, 69.4%; male: 637/1037, 61.4%; online gamers: 388/511, 76.3%) participated in a school-based GD prevention program and completed a self-report battery, which included the adapted BPNSFS-G, a validated GD scale, and ad hoc items (daily gaming time and weekly frequency). The Spanish adaptation of the BPNSFS-G was developed using the back-translation method to ensure its content validity. A structural equation modeling approach was used to test its structural, construct, convergent, and discriminant validity, as well as measurement invariance. Reliability at both the item and factor levels, along with criterion validity, was also assessed.

**Results:**

Through exploratory and confirmatory factor analyses, the scale structure was confirmed, validating 2 separate measures: a 3-factor second-order model for NS and a unidimensional model for NF, both with adequate internal consistency (NS: Cronbach α=0.75; NF: Cronbach α=0.77). The results also supported its validity, with NF more strongly associated with GD (*r*=0.49), and NS more closely related to gaming time (*r*=0.25-0.36). The scales functioned similarly across sex and developmental stage groups, but not across gaming modalities. Notably, NS showed significant differences across all subgroups, especially between boys and girls (*t*_759.31_=8.28; *P*<.001; *g*=0.55) and between online and offline gaming modes (*t*_178.47_=5.13; *P*<.001; *g*=0.58), with no meaningful differences found for NF.

**Conclusions:**

This study provides initial evidence for the validity and reliability of the Spanish version of the BPNSFS-G for adolescents. The resulting Youth Gaming Experience Scales (Youth Satisfying Gaming Experience Scale and Youth Frustrating Gaming Experience Scale) are brief, theoretically grounded, and empirically supported instruments for assessing in-game psychological need satisfaction and frustration. Given NF’s potential as a risk factor, these scales have a strong potential to explore self-determination theory–based mechanisms underlying the motivational etiology of GD and to advance both its assessment and prevention in adolescent populations.

## Introduction

Gaming represents one of the most widespread forms of virtual leisure worldwide. Its relevance is reflected in a global gamer population exceeding 3 billion gamers [1]. In Spain, 77% of young people aged 6 to 14 years and 85.1% of adolescents aged 14 to 18 years engage in gaming regularly [2,3]. Although gaming has transcended age barriers and become a pastime across generations [4], it is during childhood that both traditional (nondigital) and digital gaming habits are formed. Both digital and nondigital gaming share a core feature: their capacity to engage players through intrinsic motivation [5]. However, the key distinction between both categories lies in the potential maladaptive use of digital gaming, which can develop into an addictive disorder called gaming disorder (GD). GD is recognized as a persistent gaming behavior that significantly impairs psychosocial functioning in both the *DSM-5-TR* (*Diagnostic and Statistical Manual of Mental Disorders* [Fifth Edition, Text Revision]) and the *ICD-11* (*International Classification of Diseases, 11th Revision*) psychiatric classifications. However, while the *DSM-5-TR* classifies GD as a condition warranting further research, the *ICD-11* recognizes it as a formal disorder [6,7]. Despite these nosological differences, this study adopts the American Psychiatric Association’s *DSM-5* (*Diagnostic and Statistical Manual of Mental Disorders* [Fifth Edition]) based framework for GD, which remains widely used in research and clinical settings [8].

GD represents a growing public health concern, affecting approximately 3% of gamers worldwide [9] and 4.6% of young male gamers [10]. In Spain, its prevalence among adolescents aged 14-18 years is 7.1%, rising to 11.3% among male gamers compared to 2.7% among female gamers [3]. This higher prevalence among adolescent male gamers is consistent with established risk factors for GD, such as male sex and adolescence [11], along with gaming-related characteristics like online gaming [12]. Moreover, a growing body of research highlights gaming motivation as a significant factor in the development of GD [13]. Specifically, the self-determination theory (SDT) [14] provides a comprehensive framework for understanding the etiology of GD in youths [15].

SDT posits that gaming facilitates the fulfillment of the 3 fundamental psychological needs—autonomy (volition and personal agency), competence (sense of efficacy), and relatedness (social connection)—both in-game and in real-life [14,16]. Building on SDT, the need density hypothesis [17] proposes that GD may develop when in-game need satisfaction (NS) significantly exceeds real-life need satisfaction [18].

Although widely accepted [19,20], empirical support for this framework is inconsistent. Some studies could not replicate the expected effects of the imbalance between NS and real-life need satisfaction [21], while others found no direct association between NS and GD [18,22]. According to the need density hypothesis, recent research suggests that real-life need frustration may be a stronger predictor of GD than low real-life need satisfaction [22,23], especially when combined with high NS [18,24,25]. However, gaming environments can also frustrate these needs—defined as the active thwarting rather than merely the absence of satisfaction [24,26]—an aspect not yet fully addressed by SDT [27,28].

Originally, SDT posits that NS is linked to positive gaming outcomes, such as enjoyment, intrinsic motivation, vitality, engagement, and well-being [14,16,29-31], while in-game need frustration (NF) is associated with dysfunctional and obsessive use patterns that contribute to addictive behaviors, such as GD [24,32]. Notably, both real-life need frustration and NF have been linked to GD [22,23,25,28]. However, research on NF has received considerably less attention compared to real-life need frustration [25,27,28].

This gap is not unexpected, as SDT’s motivational model of gaming has historically centered on the study of NS [16,30], with more limited theoretical development concerning NF [27]. This emphasis on NS is also reflected in the availability of validated instruments designed to measure NS—such as the Player Experience of Need Satisfaction (PENS) [16], the Game Experience Questionnaire [33], and the Ubisoft Perceived Experience Questionnaire [34]—but not in the assessment of NF. To date, the prevailing approach has assessed NF in conjunction with NS, typically through adapted versions of the Basic Psychological Need Satisfaction and Frustration Scale (BPNSFS) [35] for gaming contexts [25,36]. However, most studies have used ad hoc adaptations without evaluating their psychometric properties [37]. This is particularly noteworthy, given that a validated version of the Basic Psychological Need Satisfaction and Frustration Scale for Gaming (BPNSFS-G) specifically designed to assess NS and NF is already available [28].

The current state of the literature reveals several critical gaps that warrant further investigation. First, research on NF and its relationship with GD remains both limited and methodologically inconsistent. Second, most studies have focused on university students or adult samples [25,28,36,37], while only a few studies have examined NS or NF using adolescent samples [20,21]. Finally, the available instruments to assess these constructs have been developed exclusively in English, restricting the generalizability of findings.

In response, this study aimed to translate the BPNSFS-G into Spanish, adapt it for the adolescent population, and validate its psychometric properties in a sample of adolescent gamers. Specifically, the study assessed the scale’s structural validity, internal consistency, as well as construct and criterion validity. Once the psychometric adequacy was established, we tested two hypotheses based on the current state of literature (1) that NF would show a stronger association with GD than NS and (2) that NS would be more closely related to gaming time and frequency than NF. Additionally, we conducted measurement invariance analyses across subgroups defined by known GD risk factors such as the developmental stage (early vs middle adolescence), sex (girls vs boys), and the gaming mode (online vs offline). This approach enabled us to explore the functioning of NS and NF across different adolescent gamer subpopulations. To our knowledge, this is the first study to examine these questions.

## Methods

### Participants

Participants were recruited through convenience sampling from 27 schools in Valencia, where the “Gamer” GD prevention program [38] was conducted from October 2022 to May 2023. The program lasts 3 weeks with 1 session per week. A pre- and postevaluation was conducted, but this study focuses exclusively on the baseline assessment, using a cross-sectional design. The questionnaires were paper-based and self-administered in groups under the supervision of prevention specialists.

The program targeted students aged 10 to 15 years (fifth year in primary school to second year in secondary school). Participants were classified into early adolescence (10-12 years old) and middle adolescence (13-15 years old) [39]. The inclusion criteria were no missing responses on any questionnaire item, sufficient Spanish proficiency, and identification as gamers on predefined criteria (see Demographic and Gaming Patterns Measures section for details).

Of the 1747 students evaluated, 1174 met the inclusion criteria (mean age 12.07, SD 1.23 years; early adolescents: 359/1174, 30.6% and middle adolescents: 815/1174, 69.4%), with 1037 participants reporting their sex (female: 400/1037, 38.6% and male: 637/1037, 60.6%) and 511 reporting their gaming modality (online gamers: 388/511, 75.9% and offline gamers: 123/511, 24.1%).

### Ethical Considerations

The study was approved by the University of Valencia Ethics Committee (approval H1482079199937), in accordance with institutional and national ethical standards, including the Declaration of Helsinki and Spanish regulations on biomedical research and personal data protection. Participation was voluntary and anonymous, and institutional agreement was obtained from the participating schools prior to data collection. Questionnaires used unique anonymized ID codes, with no personally identifiable information collected, and data were stored securely and accessed only by authorized research personnel. No financial compensation was provided.

### Procedure

The BPNSFS-G [28] was adapted into Spanish (Multimedia Appendix 1) using the back-translation method [40], following a 6-phase structure [41]. First, 2 independent translations of the original scale were produced in adolescent psychology and gaming motivation research, all experienced in psychometric validation, linguistic adaptation, and proficient in English. Second, these versions were reviewed and merged into a single refined Spanish version. In the third phase, a bilingual English linguist back-translated this version, which was then assessed by the expert panel for its semantic, conceptual, and cultural equivalence with the original scale.

The fourth phase involved adapting the questionnaire to the adolescent population at both linguistic and methodological levels. First, the panel made linguistic modifications to simplify terminology and improve item clarity for adolescents while maintaining the original meaning. Furthermore, the response scale was reduced from 7 to 5 points to enhance reliability within this population [42]. This modification was deemed necessary because simpler formats are more appropriate for the cognitive development–level characteristic of early adolescence [43]. In addition, item wording was adjusted to ensure applicability to both online and offline gaming experiences, referencing gamers’ experiences with the games they usually play [16], rather than restricting responses to a specific gaming modality [28].

The fifth phase consisted of a pilot study conducted in 4 schools (mean age 12.26, SD 0.91 years; girls: 92/199, 46.2%; boys: 93/199, 46.7%; nonbinary sex: 6/199, 3%; early adolescents: 49/199, 24.6%; middle adolescents: 150/199, 75.4%). Based on feedback from students, teachers, and prevention specialists, minor linguistic adjustments were made. A second pilot study was conducted in 2 additional schools (mean age 11.79, SD 1.07 years; girls: 25/56, 44.7%; boys: 31/56, 53.4%; early adolescents: 32/56, 55.2%; middle adolescents: 24/56, 41.4%), confirming the scale’s comprehensibility. The expert panel approved the final version of the instrument, confirming its content validity for use in the main study.

### Measures

#### Satisfaction and Frustration of Basic Psychological Needs During Gameplay

The BPNSFS-G [28] is a self-administered questionnaire designed to assess NS and NF, each conceptualized as a second-order construct composed of 3 first-order dimensions: autonomy, competence, and relatedness in-game needs.

The original version comprises 15 items rated on a 7-point Likert scale. For this study, the scale was adapted to a 5-point Likert format (0=strongly disagree to 4=strongly agree). In the original questionnaire, each item ended with the phrase “in my current favorite online game.” In the adapted version, this was changed to “the games you normally play” as an introductory statement to broaden its applicability beyond online gaming.

The satisfaction subscale consists of 7 items, selected from the PENS [16]. Higher scores indicate that video games are perceived as a source of need fulfillment within the gaming context. The subscale includes 3 items for autonomy (eg, “I feel that the decisions I make are the ones I really want to make”), 2 for competence (eg, “I feel skilled at what I do”), and 2 for relatedness (eg, “I feel close to the people who are important to me in the game”).

The frustration subscale consists of 8 items, adapted from the BPNSFS [35]. Higher scores indicate that video games are perceived as a context where psychological needs are thwarted. The subscale includes 3 items for autonomy (eg, “I feel forced to do many things that I would not do if I had a choice”), 3 for competence (eg, “I feel insecure about my abilities in the game”), and 2 for relatedness (eg, “I feel excluded from the group I want to belong to in the video game”).

#### Gaming Disorder

GD was assessed using an abbreviated version of the Video Game Dependency Test [44], adapted to the DSM-5 and ICD-11 diagnostic criteria for GD [45]. This self-administered, paper-based questionnaire consists of 10 items assessing gaming-related behaviors (eg, “I spend less time doing other activities because video games take up a significant part of my time”) and their impact over the past 12 months, rated on a 5-point Likert scale (0=strongly disagree to 4=strongly agree). The total score ranges from 0 to 40, with higher scores indicating greater symptom severity.

This version of the Video Game Dependency Test has been adapted for Spanish-speaking adolescents, demonstrating adequate internal consistency in this study (Cronbach α=0.85) comparable to the original validation (Cronbach α=0.874) [45].

#### Demographic and Gaming Pattern Measures

This set of ad hoc items was designed to assess participants’ sociodemographic characteristics and gaming patterns. Sociodemographic data comprised age, sex, and school grade. Gaming behavior was measured through daily gaming time and weekly gaming frequency, using 3 self-reported items on a 5-point ordinal scale. Daily gaming time was measured separately for weekdays and weekends, with response options ranging from 1=I do not play, 2=less than 1 hour, 3=1-2 hours, 4=2-3 hours, to 5=3 or more hours. Weekly gaming frequency was evaluated with response options ranging from 1=I do not usually play, 2=1-2 days, 3=3-4 days, 4=5-6 days, to 5=every day. Higher scores on time and frequency measures indicate greater gaming engagement. These 3 items were used as inclusion criteria when participants scored ≥3 on both daily gaming time and weekly frequency. Additionally, participants reported their preferred gaming modality, indicating whether they primarily played offline or online.

### Statistical Analyses

Data analyses were conducted using SPSS Statistics (version 28; IBM Corp) and RStudio (Posit Software, PBC). Given the study’s multivariate design, structural equation modeling was used to assess factorial structure, measurement invariance, and construct validity. The diagonal weighted least squares estimation method was applied due to the ordinal scale and nonnormal distribution of the data [46].

To analyze structural validity, exploratory factor analysis (EFA) was first conducted using principal component analysis with Varimax rotation. Data adequacy was confirmed using the Kaiser-Meyer-Olkin measure (≥0.80) and Bartlett test (*P*<.001), and factors were then retained based on eigenvalues (>1) and communalities (*h*^2^≥0.30). Second, confirmatory factor analysis (CFA) tested the factor structure derived from the EFA against competing theoretical models, with model fit evaluated using chi-square (*P*>.001), comparative fit index (CFI; >0.95), root-mean-square error of approximation (RMSEA; <0.06), and standardized root-mean-square residual (SRMR; <0.08) [47]. Third, measurement invariance was assessed across sex (boys vs girls), the developmental stage (early vs middle adolescents), and the gaming modality (online vs offline) using a multilevel approach. This included testing configural, metric, and scalar invariance, with changes in ΔCFI (≤0.01) and ΔRMSEA (≤0.015) as criteria. For ΔSRMR, thresholds were set at ≤0.03 for metric and ≤0.01 for scalar invariance [48].

Following the selection of the final factorial model, descriptive statistics including mean, SD, skewness, and kurtosis were computed to analyze the distribution of scores. Group differences were examined using independent samples 2-tailed t tests (bilateral) with 95% CIs and Hedges g effect sizes (small≥0.2, medium≥0.5, or large≥0.8). Finally, internal consistency was assessed using Cronbach α and composite reliability (CR; ≥0.7). Item reliability was assessed using Cronbach α if an item was deleted (α–item) and the corrected item-total correlation (CITC) was 0.3 [49]. Construct and criterion validity were tested via CFA and Fisher bilateral correlations with gaming behavior indicators (daily gaming time and weekly frequency) and GD. Other indicators of internal validity were assessed using standardized factor loadings (λₑ>0.4), squared multiple correlations (*R*^2^≥0.5), and average variance extracted (AVE) to evaluate convergent validity (AVE≥0.5; CR≥0.7; and λₑ>0.5), as well as discriminant validity (AVE>*r*^2^ between factors) [50,51]. AVE and CR were manually calculated using standard formulas [51]:



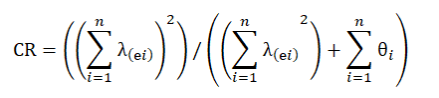





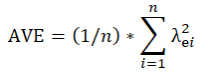



λ_e_*_i_* represents the standardized factor loading of item *i*, *n* is the number of items in the construct, and θ*_i_* refers to the standardized measurement error associated with each item.

## Results

### Factorial Analyses

The objective of this section was to examine the internal structure and measurement invariance of the scale. The scale was originally designed as a unified instrument to assess both NS and NF. The results of exploratory, confirmatory, and multigroup factor analyses supported a differentiated structure, leading to the validation of 2 independent measures: a 3-factor second-order model for NS and a unidimensional model for NF.

First, the internal structure of the questionnaire was subjected to an EFA. The results of the EFA indicated a 2D structure with 2 first-order factors composed of NS and NF (Kaiser-Meyer-Olkin measure=0.812; *χ*^2^_105_=3799.0; *P*<.001). The first factor (NS) obtained an eigenvalue of 2.26 and explained 22.66% of the variance, while the second factor (NF) obtained an eigenvalue of 3.4 and explained 17.73% of the variance. The principal component analysis excluded items with low values in their communalities, including: “I am not clear if I can do things well in the video game” (competence frustration=0.17) and “I feel that what I choose in video games really expresses how I am” (autonomy satisfaction=0.27).

Second, a CFA was performed across the predefined models to identify the best fit for the data. The structure obtained from the EFA was rejected, despite its consistency with the theoretical model (*χ*^2^_64_=863.9; *P*>.001; CFI=0.82; RMSEA=0.11; 90% CI 0.097-0.12; SRMR=0.09). We then tested 2 factorial structures defined in the literature through competitive CFA. Both models included 6 factors for in-game needs, but model 1 was defined by a second-order structure [28] and model 2 by a first-order structure [35].

Model 2 (*χ*^2^_75_=617.4; *P*>.001; CFI=0.92; RMSEA=0.072; 90% CI=0.064-0.079; SRMR=0.067) performed better than model 1 (*χ*^2^_83_=853.1; *P*>.001; CFI=0.89; RMSEA=0.088; 90% CI 0.076-0.09; SRMR=0.084), which improved after eliminating the problematic items identified in EFA (model 1: *χ*^2^_83_=853.1; *P*>.001; CFI=0.88; RMSEA=0.088; 90% CI 0.076-0.09; SRMR=0.084; and model 2: *χ*^2^_75_=617.4; *P*>.001; CFI=0.92; RMSEA=0.072; 90% CI 0.064-0.079; SRMR=0.067). However, both models were rejected because of the RMSEA values.

Since the original questionnaire measured NS independently (PENS) [16,28], a second-order model with 3 first-order dimensions for both NS and NF (model 3) was tested. This decision followed the unsatisfactory results of the CFA, where an attempt to declare NS and NF as independent second-order factors resulted in a poor fit (*χ*^2^_84_=766.1; *P*>.001; CFI=0.88; RMSEA=0.081; 90% CI 0.077-0.088; SRMR=0.09), even after excluding the problematic items (*χ*^2^_59_=505.3; *P*>.001; CFI=0.91; RMSEA=0.081; 90% CI 0.073-0.09; SRMR=0.084).

**Figure 1:**
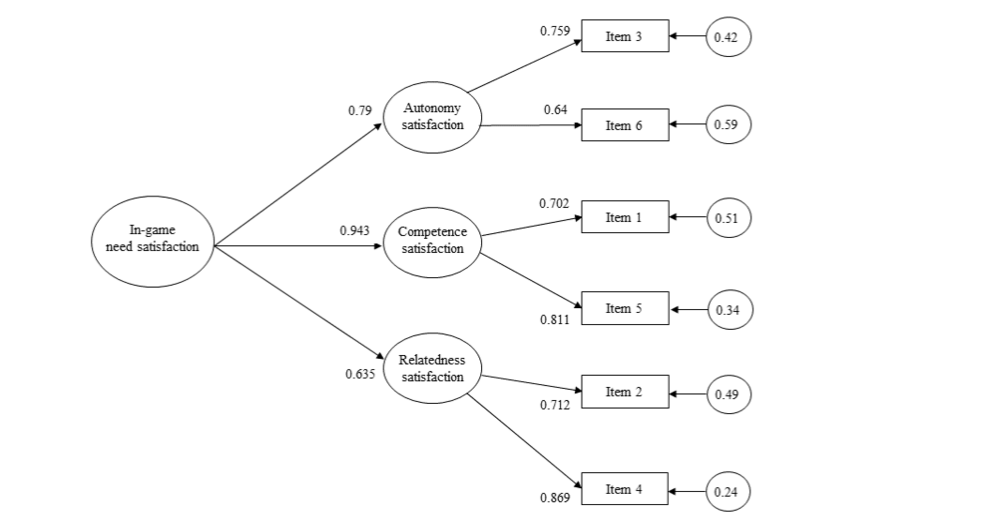
3D model of in-game need satisfaction.

The CFA results improved notably when the fit of model 3 was analyzed separately for both scales. For the NS scale, the fit approached ideal values (*χ*^2^_11_=52.3; *P*>.001; CFI=0.98; RMSEA=0.058; 90% CI 0.041-0.077; SRMR=0.029), which improved further after eliminating the autonomy satisfaction item, as it did not meet the cutoff for factor saturation (λ_e_=0.456). Additionally, the 3-factor structure without this item (*χ*^2^_6_=12.4; *P*=.05; CFI=0.99; RMSEA=0.052; 90% CI 0.028-0.079; SRMR=0.015) was superior to the unidimensional structure (*χ*^2^_14_=425.7; *P*>.001; CFI=0.81; RMSEA=0.16; 90% CI 0.15-0.17; SRMR 0.077). Figure 1 shows the final structure of the NS scale.

**Figure 2:**
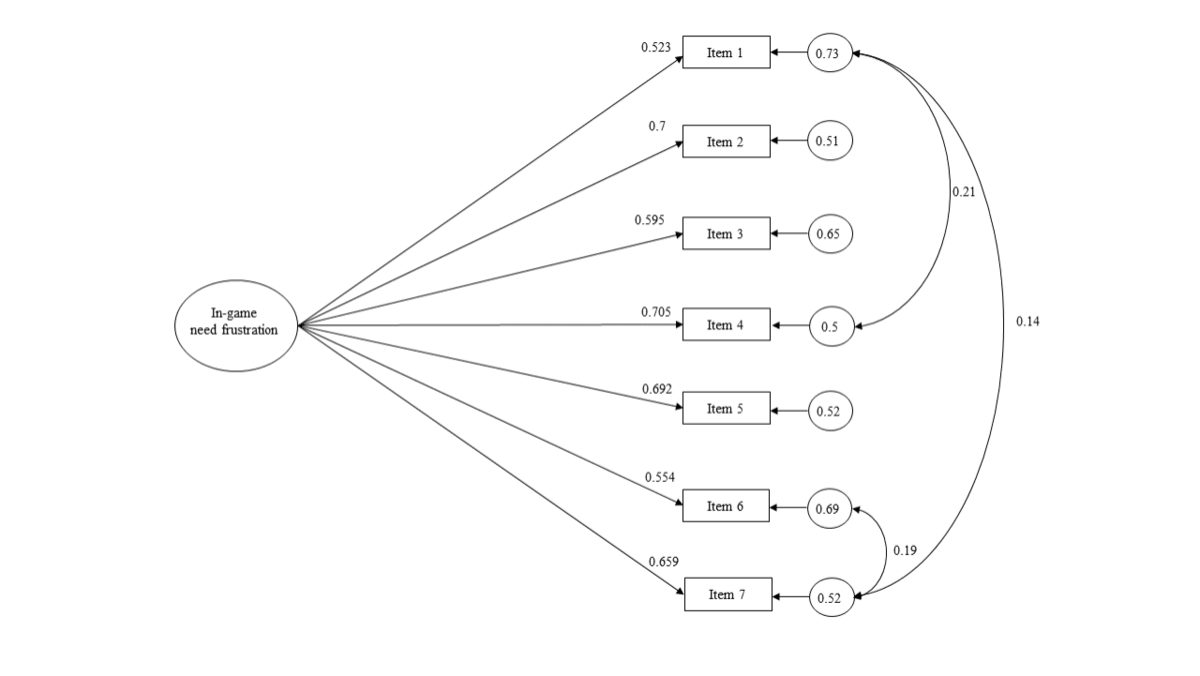
Unidimensional model of in-game need frustration.

Conversely, the results of model 3 on the NF scale were quite satisfactory but not enough because of RMSEA (*χ*^2^_17_=87.4; *P*>.001; CFI=0.957; RMSEA=0.076; 90% CI 0.061-0.092; SRMR=0.033) and a problematic item from the competence frustration factor (λ_e_=0.389). Analyses were replicated, removing this item, although the results were lower (*χ*^2^_11_=67.8; *P*>.001; CFI=0.962; RMSEA=0.087; 90% CI 0.069-0.107; SRMR=0.033). Finally, a unidimensional model was tested (*χ*^2^_20_=99.1; *P*>.001; CFI=0.952; RMSEA=0.075; 90% CI 0.061-0.089; SRMR=0.036), which did improve by removing this item (*χ*^2^_14_=82.3; *P*>.001; CFI=0.96; RMSEA=0.08; 90% CI 0.064-0.098; SRMR=0.036), reaching very satisfactory values when relating the error covariances of items 1, 4, 6, and 7 of the scale (*χ*^2^_11_=32.8; *P*>.001; CFI=0.986; RMSEA=0.053; 90% CI 0.032-0.074; SRMR=0.023). Figure 2 shows the final structure of the NF scale.

Finally, CFA supported a 3D second-order structure for NS and a unidimensional structure for NF. After confirming different structures for NS and NF, its measurement invariance was tested. The analyses showed that both scales met the invariance criteria for developmental stage (Table 1) and sex (Table 2) groups, but not for the modality of play (Table 3). Specifically, the NF scale met both levels of invariance for the measurement of girls and boys as well as early and middle adolescents, although the NS scale only reached weak invariance for the developmental stage.

**Table 1:** Invariance of in-game need satisfaction (NS) and in-game need frustration (NF) based on developmental stage.

Groups			Developmental stage					*P* value			CFI^a^ (Δ)			RMSEA^b^ (Δ)			SRMR^c^ (Δ)
			Early adolescence (358/717), chi-square (*df*)		Middle adolescence (359/717), chi-square (*df*)												
**NS**																	
	Configural	5.6 (12)		18.4 (12)		.02			0.994 (—^d^)			0.053 (—)			0.023 (—)		
	Metric	9 (15)		18.9 (15)		.02			0.994 (0)			0.049 (0.004)			0.031 (0.008)		
	Scalar	9.5 (30)		18.3 (30)		.58			1 (0.006)			0.001 (0.048)			0.024 (0.007)		
**NF**																	
	Configural	12.9 (22)		43.6 (22)		.001			0.984 (—)			0.066 (—)			0.039 (—)		
	Metric	17.0 (28)		37.5 (28)		.002			0.988 (0.004)			0.051 (0.015)			0.045 (0.006)		
	Scalar	26.5 (48)		50.7 (48)		.005			0.986 (0.002)			0.041 (0.01)			0.041 (0.004)		

^a^CFI: comparative fit index.

^b^RMSEA: root-mean-square error of approximation.

^c^SRMR: standardized root-mean-square residual.

^d^Not applicable.

**Table 2:** Invariance of in-game need satisfaction (NS) and in-game need frustration (NF) based on sex.

Groups		Sex			*P* value		CFI^a^ (Δ)		RMSEA^b^ (Δ)		SRMR^c^ (Δ)
		Girls (400/800), chi-square (*df*)	Boys (400/800), chi-square (*df*)								
**NS**											
	Configural	8.3 (12)	18.7 (12)	.008		0.992 (—^d^)		0.056 (—)		0.028 (—)	
	Metric	12.0 (15)	27.4 (15)	.001		0.988 (0.04)		0.064 (0.008)		0.038 (0.01)	
	Scalar	30.2 (30)	50.5 (30)	.001		0.974 (0.014)		0.065 (0.001)		0.04 (0.004)	
**NF**											
	Configural	12.9 (22)	20.7 (22)	.054		0.995 (—)		0.036 (—)		0.028 (—)	
	Metric	16.6 (28)	22.6 (28)	.078		0.996 (0.001)		0.032 (0.004)		0.034 (0.006)	
	Scalar	35.7 (48)	38.6 (48)	.009		0.99 (0.006)		0.037 (0.005)		0.034 (0)	

^a^CFI: comparative fit index.

^b^RMSEA: root-mean-square error of approximation.

^c^SRMR: standardized root-mean-square residual.

^d^Not applicable.

**Table 3:** Invariance of in-game need satisfaction (NS) and in-game need frustration (NF) based on the gaming modality.

Groups			Gaming modality				*P* value		CFI^a^ (Δ)		RMSEA^b^ (Δ)		SRMR^c^ (Δ)
			Online (123/246), chi-square (*df*)		Offline (123/246), chi-square (*df*)								
**NS**													
	Configural	5.3 (12)		4.9 (12)		.60		1 (—^d^)		0 (—)		0.029 (—)	
	Metric	9.5 (15)		9.7 (15)		.21		0.994 (0.006)		0.047 (0.047)		0.047 (0.018)	
	Scalar	15.0 (30)		13.8 (30)		.53		1 (0.006)		0 (0.047)		0.042 (0.005)	
**NF**													
	Configural	12.0 (22)		22.8 (22)		.04		0.985 (—)		0.069 (—)		0.052 (—)	
	Metric	26.1 (28)		28.6 (28)		.002		0.968 (0.017)		0.088 (0.019)		0.075 (0.033)	
	Scalar	23.2 (48)		34.2 (48)		.17		0.989 (0.021)		0.04 (0.048)		0.06 (0.015)	

^a^CFI: comparative fit index.

^b^RMSEA: root mean-square-error of approximation.

^c^SRMR: standardized root-mean-square residual.

^d^Not applicable.

### Descriptive Statistics and Differences Between Groups

Overall, NS was higher than NF, which had notably low values, although NS scores showed greater variability (Table 4). Specifically, autonomy (followed by competence) showed the highest satisfaction, while relatedness had the lowest.

**Table 4:** Description of the scores of the scales of basic need satisfaction and frustration in the gaming context^a^.

Scales and items					Mean (SD)			Skewness			Kurtosis
“**While playing the video games I normally play ...”**											
	**NS^b^**										
		1. ... I am confident that I am good at playing.	2.79 (0.98)			–0.49			–0.12		
		2. ... I feel close to the people who are important to me in the game.	1.86 (1.38)			0.37			–1.21		
		3. ... I feel like I have a choice and a sense of freedom with the things I do in the game.	2.8 (1.21)			–0.77			–0.37		
		4. ... I feel close to the people I give to and receive support from in the game.	1.96 (1.38)			–0.07			–1.21		
		5. ... I feel skilled at what I do.	2.62 (1.20)			–0.64			–0.42		
		6. ... I feel that the decisions I make are the ones I really want to make.	2.75 (1.28)			–0.8			–0.39		
	**NF^c^**										
		1. ... most of the things I do I feel I have to do out of obligation.	0.92 (1.12)			10.04			0.17		
		2. ... I feel excluded from the group I want to belong to in the game.	0.7 (10.0)8			1.53			1.54		
		3. ... when I make mistakes while playing, I feel like a failure.	0.8 (1.1)			1.35			10.01		
		4. ... I feel forced to do many things that I would not do if I had a choice.	1.06 (1.21)			0.87			–0.28		
		5. ... I feel that the people who are important to me in the video game are cold and distant to me during the game.	0.8 (1.11)			1.29			0.78		
		6. ... I feel insecure about my abilities in the game.	0.84 (10.07)			1.13			0.47		
		7. ... I feel pressured to do too many things in the game.	0.83 (1.14)			1.26			0.64		
**Total scores**											
	NS			14.5 (5.6)			–0.37			–0.14	
	Autonomy			5.56 (20.09)			–0.68			–0.26	
	Competence			5.41 (1.89)			–0.52			–0.13	
	Relatedness			3.82 (2.43)			0.01			–10.01	
	NF			5.95 (50.04)			0.79			0.01	

^a^Originally translated from Spanish (Multimedia Appendix 1).

^b^NS: in-game need satisfaction.

^c^NF: in-game need frustration.

The scoring trend of the different groups was similar to the overall sample scores (Tables 5-7). The highest NS scores were obtained by the online gamers’ subgroup, followed by the male participants, and finally by the early adolescents. These same populations also obtained higher scores on the NF, although in this case, the subgroup that presented greater frustration was the early adolescents, followed by the online gamers, and finally the male participants.

Effect sizes indicated that the population variables used to define gamer groups based on needs were relevant for sex and the game modality but not for the developmental stage. However, while the effect size was medium-high in the case of the NS factors, NF had a low or no effect. Therefore, it seems that these personal variables had a greater impact on the experience of NS than on NF.

**Table 5:** Differences between adolescent groups on the scores of in-game need satisfaction (NS) and in-game need frustration (NF) scales.

	Developmental stage		*t* test (*df*=1172)	*P* value^a^	95% CI	*g*
	Early adolescence (359/1174), mean (SD)	Middle adolescence (815/1174), mean (SD)				
AS^b^	5.57 (2.05)	5.56 (2.12)	0.1	.92	–0.25-0.27	–0.12
CS^c^	5.64 (1.78)	5.31 (1.93)	2.7	.007	0.09-0.56	0.05
RS^d^	4.14 (2.43)	3.67 (2.42)	3.04	.002	0.17-0.77	0.07
NS	17.15 (5.71)	16.23 (5.53)	2.6	.01	0.23-1.62	0.04
NF	6.43 (5.08)	5.75 (5.01)	2.14	.03	0.06-1.31	0.01

^a^The *P* value reflects the 2-tailed hypothesis test.

^b^AS: autonomy satisfaction.

^c^CS: competence satisfaction.

^d^RS: relatedness satisfaction.

**Table 6:** Differences between sex groups on the scores of in-game need satisfaction (NS) and in-game need frustration (NF) scales.

	Sex		*t* test (*df*)	*P* value^a^	95% CI	*g*
	Girls (400/1037), mean (SD)	Boys (637/1037), mean (SD)				
AS^b^	5.21 (2.25)	5.93 (1.86)	5.37 (730.36)	<.001	0.46-0.99	0.36
CS^c^	4.72 (1.9)	5.86 (1.73)	9.77 (788.67)	<.001	0.91-1.4	0.64
RS^d^	3.24 (2.44)	4.31 (2.36)	7.02 (1035)	<.001	0.77-1.4	0.45
NS	14.9 (5.8)	17.82 (5.04)	8.28 (759.31)	<.001	2.2-3.6	0.55
NF	5.53 (4.93)	5.98 (4.98)	1.46 (1035)	.15	0.17-1.07	0.09

^a^The *P* value reflects the 2-tailed hypothesis test.

^b^AS: autonomy satisfaction.

^c^CS: competence satisfaction.

^d^RS: relatedness satisfaction.

**Table 7:** Differences between gaming modality groups on the scores of in-game need satisfaction (NS) and in-game need frustration (NF) scales.

	Modality			*t* test (*df*)		*P* value^a^		95% CI		*g*
	Online (388/511), mean (SD)	Offline (123/511), mean (SD)								
AS^b^	6.13 (1.68)	5.79 (1.89)	1.91 (590)		.05		–0.01 to 0.7		0.2	
CS^c^	5.94 (1.63)	5.27 (1.8)	3.67 (189.65)		<.001		0.33 to 1.01		0.4	
RS^d^	4.82 (2.27)	3.25 (2.55)	6.1 (186.34)		<.001		1.06 to 2.08		0.67	
NS	18.81 (4.58)	16 (5.51)	5.126 (178.47)		<.001		1.73 to 3.89		0.58	
NF	6.15 (5.02)	5.27 (4.97)	1.7 (509)		.09		–0.14 to 1.9		0.18	

^a^The *P* value reflects the 2-tailed hypothesis test.

^b^AS: autonomy satisfaction.

^c^CS: competence satisfaction.

^d^RS: relatedness satisfaction.

### Psychometric Evaluation of the Constructs

To ensure the psychometric properties of the scale’s factors, we analyzed the main reliability and validity indicators (Table 8) and their relationship with gaming patterns and GD (Table 9). Item correlation analyses revealed higher correlations between items within the same factor than between items of different factors for the NS scale, but not for the NF scale. This supports the multifactorial and single-factor structures, respectively. Both scales showed adequate discriminant validity through the squared correlational values (*r*^2^) between factors (autonomy-competence=0.179; autonomy-relatedness=0.084; competence-relatedness=0.169 [NS]; NS-NF=0.012).

**Table 8:** Psychometric study of in-game psychological needs factors.

		NS^a^				NF^b^
		AS^c,d^	CS^e,f^	RS^g,h^	TS^i,j^	TS^k^
**AS**						
	*r*^l^ (95% CI)	1	0.47 (0.43 to 0.52)	0.32 (0.27 to 0.37)	0.76 (0.73 to 0.78)	–0.076 (–0.133 to –0.019)
	*P* value	>.99	<.001	<.001	<.001	.009
**CS**						
	*r* (95% CI)	0.47 (0.43 to 0.52)	1	0.42 (0.37 to 0.47)	0.78 (0.76 to 0.81)	0.061 (0.004 to 0.118)
	*P* value	<.001	>.99	<.001	<.001	.04
**RS**						
	*r* (95% CI)	0.32 (0.27 to 0.37)	0.42 (0.37 to 0.47)	1	0.78 (0.76 to 0.81)	0.219 (0.164 to 0.273)
	*P* value	<.001	<.001	>.99	<.001	<.001
**TS**						
	*r* (95% CI)	0.76 (0.73 to 0.78)	0.78 (0.76 to 0.81)	0.78 (0.76 to 0.81)	1	0.098 (0.41 to 0.154)
	*P* value	<.001	<.001	<.001	>.99	<.001

^a^NS: in-game need satisfaction.

^b^NF: in-game need frustration.

^c^AS: autonomy satisfaction.

^d^Cronbach α=0.58; composite reliability=0.58; average variance extracted=0.49; *R*^2^=0.62.

^e^CS: competence satisfaction.

^f^Cronbach α=0.66; composite reliability=0.73; average variance extracted=0.58; *R*^2^=0.89.

^g^RS: relatedness satisfaction.

^h^Cronbach α=0.75; composite reliability=0.68; average variance extracted=0.64; *R*^2^=0.4.

^i^TS: total score.

^j^Cronbach α=0.75; composite reliability=0.82; average variance extracted=0.57.

^k^Cronbach α=0.77; composite reliability=0.83; average variance extracted=0.41.

^l^*r*: Pearson bilateral correlations.

**Table 9:** Associations between in-game psychological needs and patterns of gaming use and problematic behavior.

			NS^a^								NF^b^
			AS^c^		CS^d^	RS^e^		TS^f^		TS	
**MGT** ^g^											
	*r*^h^ (95% CI)	0.11 (0.05-0.17)		0.18 (0.13-0.24)		0.15 (0.09-0.24)	0.19 (0.13-0.24)		0.12 (0.06-0.17)		
	*P* value	<.001		<.001		<.001	<.001		<.001		
**WGT** ^i^											
	*r* (95% CI)	0.25 (0.17-0.28)		0.31 (0.25-0.36)		0.3 (0.25-0.036)	0.36 (0.31-0.41)		0.08 (0.03-0.14)		
	*P* value	<.001		<.001		<.001	<.001		.005		
**WGF** ^j^											
	*r* (95% CI)	0.19 (0.14-0.25)		0.24 (0.18-0.29)		0.16 (0.1-0.21)	0.25 (0.19-0.3)		0.1 (0.043-0.156)		
	*P* value	<.001		<.001		<.001	<.001		<.001		
**TDV** ^k^											
	*r* (95% CI)	0.14 (0.08-0.2)		0.3 (0.25-0.35)		0.35 (0.29-0.4)	0.34 (0.29-0.39)		0.49 (0.44-0.53)		
	*P* value	<.001		<.001		<.001	<.001		<.001		

^a^NS: in-game need satisfaction.

^b^NF: in-game need frustration.

^c^AS: autonomy satisfaction.

^d^CS: competence satisfaction.

^e^RS: relatedness satisfaction.

^f^TS: total score.

^g^MGT: midweek gaming daily time.

^h^*r*: Fisher bilateral bivariate correlation.

^i^WGT: weekend gaming daily time.

^j^WGF: weekly gaming frequency.

^k^TDV: Video Game Dependency Test.

Furthermore, except for the autonomy satisfaction factor, all the factors of the scales presented good convergent validity indices. Specifically, the NS factors obtained higher values compared to the NF factors. It is worth noting that all the constructs obtained good validity results represented by AVE and *R*^2^ (except for NF’s AVE and NS-relatedness for *R*^2^).

In addition, internal consistency analysis indicated that the different factors of NS and NF assess reliably and accurately (Table 8). However, the results of the autonomy satisfaction factor were the only results that did not exceed the α and CR cutoff point.

All items of both scales showed high reliability with CITC values above 0.3. In fact, CITC values ranged from 0.413 to 0.56, which showed that the totality of the items possessed excellent representativeness of the construct to which they belong. The *R*^2^ and standardized error values of items 1 (*R*^2^=0.273; ε=0.73), 3 (*R*^2^=0.355; ε=0.65), and 6 (*R*^2^=0.307; ε=0.69) of the NF scale and item 6 of the NS scale (*R*^2^=0.409; ε=0.59) did not show as adequate a measurement as the rest of the items; however, the elimination of any 1 of them would mean a decrease in overall reliability (NS: Cronbach α<0.749 and NF: Cronbach α<0.766).

After testing the psychometric characteristics of the scales, the relationship between NS and NF factors (Table 8), as well as with gaming patterns, was analyzed (Table 9). On the one hand, the NS subdimensions showed strong correlations with the total NS score. Although statistically significant, the correlation between NS and NF was very low. Among the NS dimensions, relatedness satisfaction showed the strongest association with frustration.

On the other hand, the relationship between needs and gaming patterns showed moderate correlations in the case of NS (*r*=0.187-0.359) and low or even null correlations in the case of NF (*r*=0.084-0.115). Weekend gaming time showed the highest correlations, followed by weekly frequency and weekday gaming time with NS and its first-order factors. Specifically, competence exhibited the strongest correlations (*r*=0.182-0.308).

Regarding GD, its strongest correlation was observed with NF, whereas NS also showed a significant but lower association. Notably, the correlation between NS and GD was slightly weaker than the correlation between NS and weekend gaming time, suggesting that satisfaction is more strongly associated with gaming engagement than with problematic use. In contrast, NF showed very low correlations with gaming patterns, reinforcing its specific association with GD rather than general gaming behavior. Among the satisfied needs, relatedness exhibited the strongest association with GD, followed by competence and autonomy.

## Discussion

### Principal Findings

This study aimed to adapt and validate the BPNSFS-G into Spanish for adolescents, resulting in the development of the Youth Gaming Experience Scales (in Spanish “Escalas para Jóvenes sobre la Experiencia de Juego” or EJEJ), designed to independently assess NS (Youth Satisfying Gaming Experience Scale [EJEJ-S]) and NF (Youth Frustrating Gaming Experience Scale [EJEJ-F]). The results supported the validity and reliability properties of both scales, showing that NF was more strongly associated with GD, while NS was more closely linked to gaming time. This pattern challenges the need density hypothesis capacity to explain GD by highlighting the central role of NF. Furthermore, for the first time, measurement invariance analyses enabled the empirical exploration of differences in these experiences according to personal and contextual factors widely recognized as GD risk markers. Overall, the EJEJs show both research and applied utility, contributing to the study, evaluation, early detection, and intervention design of GD from an SDT framework.

Factorial analyses compared several models proposed in the literature regarding the internal structure of psychological needs in gaming, including the original BPNSFS model, which treats both NS and NF as either first-order [35] or second-order factors [28]. None of these structures demonstrated satisfactory fit. Instead, a more parsimonious model based on 2 independent scales was confirmed, as suggested by the EFA results. Accordingly, 2 separate scales were defined to assess NS and NF. This model resembles the structure of the PENS scale [16,28] and supports the theoretical distinction between NS and NF as separate constructs, rather than as opposite ends of a single continuum [26,52]. Recent evidence supports this distinction, indicating that both dimensions may coexist within the gaming experience [19,24,28]. While NS and NF are typically negatively correlated in real-life contexts [35], this association does not seem to hold in gaming environments [25,28]. These findings suggest that psychological needs in video games may be simultaneously satisfied and frustrated [19].

The EJEJ-F showed a unidimensional structure, failing to differentiate between the frustration of autonomy, competence, and relatedness needs. This contrasts with the NS dimension in our study, which did show a clear 3-factor structure, in line with previous findings [28,37]. From a methodological perspective, this outcome may be influenced by factors such as the linguistic adaptation, response format, or the developmental characteristics of the adolescent sample [41]. Theoretically, it may also reflect the still-limited conceptual development of NF in gaming research. While recent models have proposed a dimensional structure for NF analogous to NS [27], our findings did not support this configuration. Nonetheless, both scales demonstrated adequate internal consistency, consistent with prior research [28], supporting the reliability of the instrument for assessing the overall construct of NF, as well as the global and specific dimensions of NS.

Regarding validity, the EJEJ-S and EJEJ-F showed distinct patterns of association with external gaming-related variables, supporting the questionnaire’s convergent and criterion validity. In line with the hypotheses, NF showed a stronger association with GD, while NS was more closely related to gaming time and frequency. This pattern aligns with SDT, where NF is conceptualized as a form of controlled motivation—driven by rigid and external regulation—linked to compulsive and dysregulated gaming behavior [27,32], while NS is associated with more autonomous and intrinsically regulated gaming, typically reflected in positive engagement indicators such as gaming time [16,29-31]. These findings highlight the EJEJ’s sensitivity to differentiate between adaptive and maladaptive gaming.

These findings, along with previous studies questioning the direct link between NS and GD [18,22], raise 2 important implications. First, the need density hypothesis [17], which suggests that GD results from an imbalance between real-life need satisfaction and NS, is called into question. Our results challenge the core assumptions of this hypothesis, highlighting NF—rather than NS—as a stronger and independent indicator of GD [22,23] in both adolescents and adults [25,28].

Second, the weak association observed between NF and gaming time contributes to the growing evidence challenging the use of gaming time as a primary diagnostic criterion for GD [53,54]. While gaming time has traditionally been viewed as a sign of “tolerance” in addiction models [6,55], several studies have questioned its validity as a standalone marker of pathology [12,56,57]. Our findings support this perspective, suggesting that gaming time may reflect normative engagement more than dysregulated use and is likely modulated by motivational factors—such as NS—or by broader regulatory processes [22,23,58,59]. This reinforces recent proposals to integrate motivational variables like NF into diagnostic models to better distinguish between intensive, but healthy, and truly problematic gaming [19,56,60].

In preventive contexts—especially within school-based programs [61]—these indicators can help detect motivational patterns that precede the development of GD. This supports the need for interventions that go beyond limiting gaming time and promote healthier psychological engagement [62], for which SDT has proven to be an effective framework in both clinical and educational contexts [63-65]. Given their sensitivity to problematic gaming patterns, the EJEJs may contribute to early risk detection and to the development of SDT-based interventions.

Supporting this applied potential, the measurement invariance analyses confirmed the factorial equivalence of the EJEJ across sex and the adolescence stage—2 key variables consistently identified as risk factors for GD [11,12]. Previous studies have explored psychological NS and NF across gaming characteristics, such as gaming genres in the BPNSFS-G [28] or level of generality of the need experience (game session, general gaming, and particular session play) in the Basic Needs in Games Scale [37]. However, to our knowledge, no prior research has examined these experiences in relation to personal variables like the adolescent stage or sex.

These results offer novel exploratory insights into the in-game experience of psychological needs among gamer adolescent subpopulations. Younger adolescents reported higher levels of both NS and NF. This greater experiential intensity may be explained by a stronger emotional involvement with gaming at earlier developmental stages [66], as well as by an increased sensitivity to the psychological experience of NS and NF, as described in SDT for this life stage [24]. Regarding sex, male participants scored higher on NS [67], with no significant differences found in NF. Although male sex is widely recognized as a key risk factor for GD [11,12], these results suggest that NF may represent a shared vulnerability mechanism across sex, which could depend on moderating variables such as gaming motivation (eg, socializing [68]) or coping style [58,59,69]. These findings underscore the need for further research into sex-related differences in the experience of NS and NF [16,22], for which the EJEJ may offer a valuable assessment tool.

In contrast, invariance was not observed across gaming modality (online vs offline), which limits direct comparisons between these groups and suggests differences in how gamers experience their psychological needs depending on the gaming modality [30]. Notably, significant differences were found in several NS dimensions across modalities (except for autonomy), with higher scores among online gamers. This could be especially relevant for the relatedness need [16], given the greater potential of online games for real interpersonal interaction, compared to offline games, which typically involve nonhuman characters. These findings highlight the need to explore how the gaming modality affects the experience and assessment of psychological needs in different gaming contexts. Previous instruments, such as the PENS [16], did not differentiate between online and offline contexts, leading to interpretive ambiguities. More recent tools like the BPNSFS-G [28] have been developed specifically for online gaming. However, the present results suggest that the validity of these assessments may not generalize across modalities, reinforcing the need for modality-specific validation.

### Limitations

This study presents several limitations that should be considered in future research. First, the cross-sectional design limits the ability to establish causal relationships. Second, although psychometric quality was supported by multivariate analyses, future studies should implement regression models to examine predictive validity. Third, the use of a convenience sample limits the generalizability of the findings, highlighting the need for future studies to include diverse cultural contexts and settings (eg, different intervention programs). Fourth, longitudinal designs are needed to assess the direction and stability of the associations between in-game needs and GD. Fifth, test-retest reliability studies should be included to evaluate the temporal consistency of the scores, particularly in the context of intervention and prevention. Finally, the lack of invariance suggests the need to develop adapted versions of the instrument for different gaming modalities.

Additionally, the brief format is both a strength—due to its practical application—and a limitation in theoretical research. Future studies should examine the specific dynamics of autonomy, competence, and relatedness in both NS and NF [14,16,27,30], a goal for which the EJEJ-S could be appropriate but not the EJEJ-F due to its unidimensional structure. Furthermore, future research should explore the relationship between in-game and real-life need experiences across adolescent populations [25].

### Conclusions

This study provides the first validation of the Spanish version of the BPNSFS-G for adolescent populations, leading to the development of EJEJ-S and EJEJ-F. The psychometric properties of these scales were thoroughly supported, demonstrating their reliability and validity across various adolescent subpopulations, including different GD risk profiles. This robustness supports their applicability to diverse contexts, suggesting their potential utility in both intervention and prevention contexts.

By highlighting NF as a key risk factor for GD, this study reinforces the theoretical value of SDT in understanding the etiology of GD in youths. NF may serve as a predictive indicator of problematic gaming and help differentiate it from normative use, highlighting its potential for early detection and intervention. These findings underscore the relevance of SDT-based approaches for developing prevention programs that promote healthy gaming behaviors.

Although the sample’s representativeness limits the generalizability of the findings, the successful adaptation of the questionnaire broadens its applicability to a wider range of populations. Future research should include longitudinal designs, regression models, and test-retest reliability analyses to improve the predictive validity and temporal consistency of the scales. In conclusion, the EJEJs have strong potential to advance both the theoretical understanding and practical application of interventions for GD within the SDT framework.

## Appendix

Multimedia Appendix 1 Original version of Youth Gaming Experience Scales.

## Abbreviations

AVE: average variance extracted
BPNSFS: Basic Psychological Need Satisfaction Frustration Scale
BPNSFS-G: Basic Psychological Need Satisfaction Frustration Scale for Gaming
CFA: confirmatory factor analysis
CFI: comparative fit index
CITC: corrected item-total correlation
CR: composite reliability
DSM-5: Diagnostic and Statistical Manual of Mental Disorders (Fifth Edition)
DSM-5-TR: Diagnostic and Statistical Manual of Mental Disorders (Fifth Edition, Text Revision)
EFA: exploratory factor analysis
EJEJ: Youth Gaming Experience Scale
EJEJ-F: Youth Frustrating Gaming Experience Scale
EJEJ-S: Youth Satisfying Gaming Experience Scale
GD: gaming disorder
ICD-11: International Classification of Diseases, 11th Revision
NF: in-game need frustration
NS: in-game need satisfaction
PENS: Player Experience of Need Satisfaction
RMSEA: root-mean-square error of approximation
SDT: self-determination theory
SRMR: standardized root-mean-square residual
