# Supplementary material for: In-Game Need Satisfaction, Frustration, and Gaming Addiction Patterns Across Subgroups of Adolescents Through Structural Equation Modeling: Cross-Sectional and Instrument Validation Study of the Youth Gaming Experience Scales
Source: JMIR Serious Games. 2025 Nov 12;13:e63612. doi: 10.2196/63612 (PMC12658397; doi:10.2196/63612)
Supplement: Multimedia Appendix 1 [file games_v13i1e63612_app1.docx]

#### Youth Gaming Satisfaction Experience Scale (Escala sobre la Experiencia de Juego Satisfactoria; EJEJ-S)

**Mientras juego a los videojuegos que suelo jugar...**

| 1. ... tengo confianza en que soy buena/o jugando. |
| --- |
| 1. ... me siento unida/o con las personas que son importantes para mí en el videojuego. |
| 1. ... siento que puedo elegir y tengo una sensación de libertad con las cosas que hago en el juego. |
| 1. ... me siento cercano a las personas a las que doy y de las que recibo apoyo en el juego. |
| 1. ... me siento hábil con lo que hago. |
| 1. ... siento que las decisiones que tomo son las que realmente quiero hacer. |

#### Youth Gaming Frustration Experience Scale (Escala sobre la Experiencia de Juego Frustrante; EJEJ-F)

**Mientras juego a los videojuegos que suelo jugar...**

| 1. ... la mayoría de cosas que hago siento que hay que hacerlas por obligación. |
| --- |
| 1. ... me siento excluido del grupo al que quiero pertenecer dentro del videojuego. |
| 1. ... cuando cometo errores jugando me siento un/a fracasada/o. |
| 1. ... me siento forzado a hacer muchas cosas que yo no haría si pudiera elegir. |
| 1. ... siento que las personas que son importantes para mí en el videojuego son fríos y distantes conmigo en el juego. |
| 1. ... me siento insegura/o de mis habilidades. |
| 1. ... me siento presionada/o a hacer demasiadas cosas. |
